# NAD+ as a metabolic immune checkpoint in cancer

**DOI:** 10.1007/s10555-026-10393-4

**Published:** 2026-11-07

**Authors:** Reza Izadpanah, Eckhard U. Alt

**Affiliations:** 1 Applied Stem Cell Laboratory, Department of Medicine/Cardiology, Tulane University School of Medicine, New Orleans, LA USA; 2 Department of Surgery, Tulane University School of Medicine, New Orleans, LA USA

**Keywords:** NAD+ metabolism, NAMPT, Tumor immunometabolism, Immune checkpoint resistance, CD38–adenosine axis

## Abstract

Nicotinamide adenine dinucleotide (NAD+) functions in cancer as a compartmentalized signaling currency, coupling tumor cell fitness to immune competence within the tumor microenvironment (TME). In malignant cells, NAD+ sustains redox balance, poly(ADP-ribose) polymerase (PARP)-dependent DNA repair, sirtuin-mediated chromatin remodeling, and metabolic stress adaptation. In immune effector cells, the same metabolite governs mitochondrial integrity, effector persistence, and resistance to exhaustion. This duality is the basis of the explanation advanced here for why first-generation inhibitors of nicotinamide phosphoribosyltransferase (NAMPT), the rate-limiting salvage pathway enzyme, failed clinically when deployed as cytotoxic monotherapies without an immunometabolic rationale. This review synthesizes the field around a central premise: NAD+ operates as a context-dependent metabolic immune checkpoint whose therapeutic value depends on where in the tumor immune ecosystem the pathway is perturbed and whether checkpoint blockade is deployed concurrently. The therapeutic landscape is reorganized around five pharmacological classes, which differ in how far each has been tested: (i) NAMPT catalytic inhibitors, evaluated clinically and withdrawn; (ii) bifunctional NAMPT/programmed death-ligand (PD-L1) compounds and proteolysis-targeting chimera (PROTAC)-mediated NAMPT degradation, both preclinical; (iii) NAD+ precursor-based immunorestorative strategies, including ex vivo rescue of age-associated chimeric antigen receptor T (CAR-T) cell dysfunction through NMN/CD38 inhibitor combinations; (iv) adenosinergic axis targeting, in which anti-CD38 and anti-CD73 agents are approved or in trials while the proposed triple-blockade of CD38, CD73, and PD-1/PD-L1 remains untested; and (v) combinations with PARP and indoleamine 2,3-dioxygenase (IDO) inhibitors, which rest on mechanistic rationale. A tripartite pharmacological taxonomy distinguishes intracellular (iNAMPT), extracellular (eNAMPT), and vesicle-associated (vNAMPT) NAMPT as discrete drug targets. The analyses examines how TME acidification, precursor competition, and extracellular NAD+ catabolism through the CD38–CD203a–CD73 noncanonical adenosine axis reshape immune fitness, and why biomarker-guided separation of tumor-directed NAD+ depletion from immune-directed NAD+ restoration is required for rational clinical translation. Throughout, findings that are experimentally established are separated from those that remain contested or hypothetical, and the criteria under which the checkpoint designation is analytically useful, together with the conditions under which it is not, are stated explicitly.

## Introduction

Immune checkpoint blockade (ICB) has redefined the treatment of advanced malignancies, yet durable responses to anti-programmed cell death protein 1 (PD-1)/programmed death-ligand 1 (PD-L1) therapy reach only a fraction of patients [1, 2]. Primary and acquired ICB resistance are multifactorial: tumor-intrinsic defects in antigen presentation, immunosuppressive reprogramming of the tumor microenvironment (TME), and metabolic competition between malignant and immune cells for glucose, glutamine, and essential amino acids [3]. Within this framework, nicotinamide adenine dinucleotide (NAD+) acts as a pleiotropic regulator of tumor cell proliferation and immune effector function [4, 5].

In cancer, NAD+ biosynthetic networks link nutrient availability to redox control, genome maintenance, epigenetic plasticity, and cell state transitions [6, 7]. NAD+ fuels glycolysis, the tricarboxylic acid (TCA) cycle, oxidative phosphorylation, one-carbon metabolism, and antioxidant systems [3–5], but it is consumed by poly(ADP-ribose) polymerases (PARPs), sirtuins, and NAD glycohydrolases (CD38 and CD157) [8], coupling metabolic throughput to stress signaling. Abundance alone is an incomplete descriptor: compartmentalization, turnover, and route of synthesis determine whether NAD+ supports anabolic growth, DNA repair, stem-like persistence, or immune effector function. Shifts in NAD+/NADH and NADP+/NADPH ratios let cancer cells meet increased metabolic demand [9]. In breast cancer, high NAD biosynthesis activity associates with poor prognosis, increased immune infiltration, and elevated expression of immune checkpoint molecules [10]. NAD+ also contributes to immune evasion and microenvironmental remodeling favoring oncogenesis [3]. Dietary nicotinamide riboside (NR) increased brain metastasis of triple-negative breast cancer (TNBC) in mice [11], indicating that precursor supplementation is not uniformly beneficial. NAD+ also supports DNA repair, signaling, apoptosis, and stress responses [8] and sustains stemness programs in glioma cancer stem-like cells [3, 12, 13].

Nicotinamide phosphoribosyltransferase (NAMPT) is the dominant rate-limiting enzyme of the salvage pathway in many tumors, and elevated expression links to poor outcomes across solid and hematologic malignancies [4, 14]. NAMPT sustains the intracellular NAD+ pools required for sirtuin 1 (SIRT1)-dependent deacetylation that suppresses p53-mediated apoptosis, for PARP-dependent repair and replication stress tolerance, and for stemness circuits such as the E2F2-ID axis in glioblastoma (GBM) [13]. This explains why NAMPT inhibition reduces viability and sensitizes tumor cells to apoptosis [15]. Pathway redundancy is therapeutically consequential: tumors with preserved nicotinic acid phosphoribosyltransferase (NAPRT) activity escape NAMPT blockade through the Preiss–Handler route [16], and *de novo* tryptophan-derived synthesis via quinolinate phosphoribosyltransferase (QPRT) provides an additional bypass [17]. NAMPT expression is therefore uninterpretable without concurrent assessment of NAPRT status and salvage dependence.

CD38, a principal NAD+-degrading ectoenzyme, acts in a contact-dependent manner. In prostate cancer, CD38 overexpression depletes proliferative NAD+ pools sufficient to induce cell cycle arrest and inhibit cancer cell metabolism, functioning as a tumor suppressor [18–20]. Chen et al. showed that CD38 is transcriptionally upregulated on tumor cells after anti-PD-1/PD-L1 treatment, promoting acquired resistance by depleting immune cell NAD+ and feeding the noncanonical adenosine cascade [21]. Combined CD38/PD-L1 inhibition restored anti-tumor immunity in this setting [21]. NAD(P)H quinone oxidoreductase-1 (NQO1) is commonly elevated in small cell lung cancer (SCLC) [22]. High-level NQO1 protein expression correlates with lower disease-free survival and 5-year survival rates in SCLC patients [22], making it both a prognostic marker and a therapeutic target. NAD+ is therefore a recurrent axis across cancer subtypes and a broadly applicable therapeutic target [23].

NAD+ -directed oncology pharmacology has been defined by instructive failures. FK866 (APO866), the first-in-class competitive NAMPT inhibitor to reach the clinic, was terminated in phase II for cutaneous T-cell lymphoma (CTCL) owing to dose-limiting thrombocytopenia and insufficient responses [24]. CHS828 (GMX1778), a cyanoguanidine-class NAMPT inhibitor, did not advance beyond phase I in advanced solid tumors [25]. These outcomes were read as evidence against the tractability of the target. Both were deployed as cytotoxic monotherapies in unselected populations, without an immunometabolic rationale, without stratification by NAPRT status, and without combination partners exploiting the reciprocal relationship between tumor NAD+ depletion and PD-L1 upregulation [10, 26]. The failure was one of pharmacological strategy, not of target biology.

This review reframes the NAD+ therapeutic landscape from a translational pharmacology perspective. Rather than organizing the field by biological pathway, the analyses are structured around therapeutic drug classes and intervention platforms, which exposes the translational deficiencies that have limited progress. Five pharmacological classes are evaluated, a tripartite NAMPT taxonomy with drug design implications is introduced, and a biomarker-guided stratification framework is developed. Combination hypotheses are identified as such, with their evidentiary basis and principal uncertainties stated where introduced.

### Defining NAD+ as a metabolic immune checkpoint: criteria, scope, and limits

The term immune checkpoint was introduced to describe cell-surface receptor–ligand pairs, principally CTLA-4/B7 and PD-1/PD-L1, that deliver coinhibitory signals to T-cells through defined cytoplasmic signaling motifs, and whose antibody-mediated interruption releases a discrete brake on effector function [27]. NAD+ satisfies none of these structural criteria. It is a diffusible dinucleotide. It has no receptor function, no cognate ligand partner, and transmits no signal through an immunoreceptor tyrosine-based inhibitory motif. Applying the checkpoint label without a qualification conflates two distinct classes of biology, and the objection that NAD+ is more accurately described as a metabolic regulator has force.

The term is used here in a restricted functional sense, stated explicitly so the claim can be evaluated and, where appropriate, rejected. A metabolic immune checkpoint denotes a metabolic node satisfying four conditions. First, it is differentially utilized by malignant and immune cells occupying the same microenvironment, such that a single systemic intervention produces divergent and potentially opposing effects according to compartment. Second, its perturbation alters the expression or the functional consequence of a canonical checkpoint axis. An effect confined to tumor cell viability does not satisfy this condition. Third, its manipulation changes the outcome of checkpoint blockade in immunocompetent but not immunodeficient hosts, localizing the effect to the immune compartment. Fourth, it is pharmacologically addressable at a defined molecular target with agents that are approved or in clinical development.

NAD+ metabolism meets these conditions. The first is satisfied by the divergent consequences of salvage pathway manipulation in tumor cells relative to CD8 + T-cells and NK cells [26, 28, 29]. The second is satisfied by the NAMPT, ten–eleven translocation-1 (TET1), phosphorylated STAT1, interferon regulatory factor-1 (IRF1), PD-L1 epigenetic axis and by the SIRT-dependent, NF-κB-mediated regulation of surface PD-L1 following NAMPT inhibition [26, 30]. The third is satisfied most directly by the observation that NAMPT-deficient hepatoma cells formed tumors of comparable size to controls in immunodeficient mice but were restrained in immunocompetent hosts, with a corresponding increase in tumor-infiltrating lymphocyte number and cytolytic activity [26]. The fourth is satisfied by NAMPT catalytic inhibitors, anti-CD38 antibodies, CD73 inhibitors, and NAD+ precursors, all of which have entered clinical evaluation [23, 25, 31, 32].

The designation is a functional analogy, and it is used in that sense throughout. It asserts no structural kinship with PD-1 or CTLA-4 and places NAD+ in no established biological category. The criteria are also not unique to NAD+. Tryptophan catabolism through IDO1, arginine depletion through arginase, and lactate accumulation through glycolytic export each satisfies the same four conditions, and IDO1 has been described as a metabolic checkpoint on precisely these grounds for more than a decade [33–36]. The framework is therefore a specific instance of a general class. What distinguishes the NAD+ node is the density of druggable enzymes along one pathway, the bidirectionality of useful intervention, and the existence of NAMPT in intracellular, extracellular, and vesicle-associated forms with non-overlapping functions.

The value of the analogy is that it constrains experiment. Describing NAD+ as a metabolic regulator carries no commitment about how an intervention should be tested. Describing it as a metabolic immune checkpoint does. It requires that the benefit of a NAD+ -directed agent be lost in an immunodeficient host, that it depend on concurrent checkpoint blockade, and that it be read out on immune endpoints. No first-generation trial was designed to meet them. The analogy is falsifiable on the same terms: a trial meeting all three conditions that showed no immune-dependent benefit would defeat it.

Recent syntheses treat nutrient competition, waste metabolite accumulation, and cofactor availability as one interacting system [33]. Within it, NAD+ is distinguished by tractability. The claim that NAD+ -directed intervention will succeed where other metabolic strategies have failed would require evidence that does not yet exist. The argument advanced here is narrower and testable: that first-generation NAMPT inhibitors failed for reasons of trial design.

## NAD+ in the tumor microenvironment: metabolic ecology, NAMPT biology, and immune vulnerability

### Metabolic competition and immune cell NAD+ insufficiency

NAD+ dynamics in the tumor microenvironment reflect metabolic demand and spatial competition, and malignant, stromal, and immune cells do not access the shared pool equally. The TME combines hypoxia, low pH, nutrient deficiency, and immunosuppression, which modulates metabolic pathways in malignant and nonmalignant cells alike [37]. It also contains immune and stem/progenitor populations, extracellular matrix, and signaling molecules [38]. Multiple biochemical pathways in these populations depend on intracellular NAD [33], and oxidative stress intrinsic to angiogenesis is modulated by NAD availability [37]. Malignant metabolism reflects driver mutations, tissue of origin, and local nutrient supply [39]. Reprogramming shifts flux toward glycolysis, generating ATP, NADPH, NADH, and biosynthetic precursors [40]. Glycolysis releases lactic acid, acidifying the TME [41, 42], which suppresses NAD production and impairs the metabolic fitness of stromal and immune cells [39, 42]. NAD+ depletion in the TME is therefore an active ecological process: malignant cells preserve biosynthetic flexibility while cytotoxic lymphocytes, natural killer (NK) cells, and antigen-presenting cells lose mitochondrial reserve and functional durability. Cancer-associated fibroblasts (CAFs) compound this through glycolytic activity and lactate production that supports tumor growth while deepening the disadvantage of infiltrating immune cells.

The mechanism connecting lactate to lymphocyte dysfunction is more specific than pH stress. Quinn et al. showed that lactate limits T-cell proliferation through the NAD(H) redox state: lactate import raises the cytosolic NADH to NAD+ ratio, and the resulting reductive stress constrains the NAD+ -dependent dehydrogenase reactions of aspartate and nucleotide synthesis [35]. Restoring NAD+ regeneration relieved the block. Lactate constrains the same population by a second route, disrupting glucose transporter GLUT10-dependent mitochondrial function required for CD8 + T cell activation [43]. These two mechanisms would respond differently to NAD+ -directed intervention, which identifies redox poise as the proximate constraint on lymphocyte expansion and predicts that raising total NAD+ without restoring the NAD+ to NADH ratio will fail.

## The NAD+ biosynthetic network: routes, compartments, and consumers

**Fig. 1:**
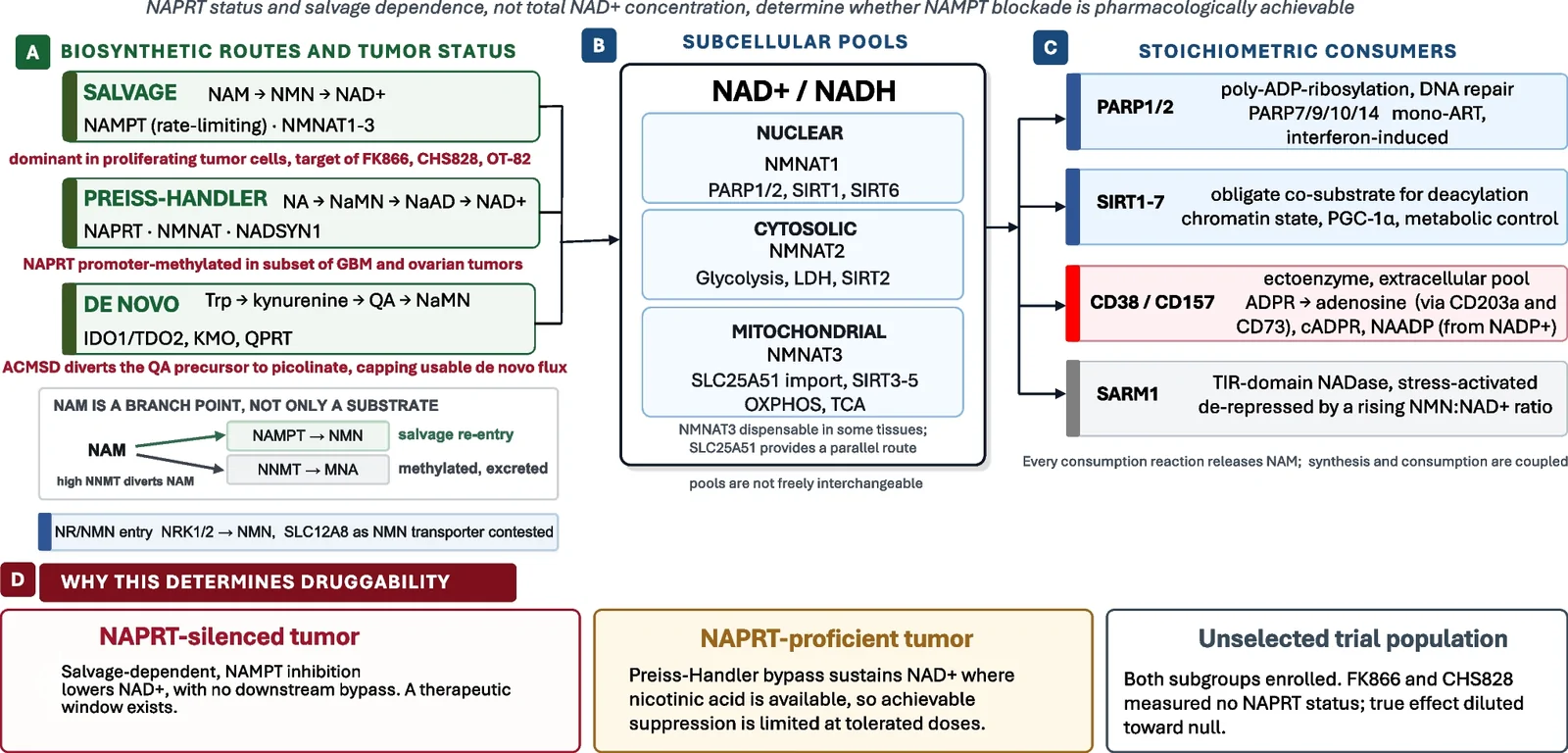
NAD+ supply, compartmentation, and consumption in the tumor cell. **a** Three biosynthetic routes converge on a common product but differ in tumor distribution. Salvage through NAMPT is rate-limiting and dominant in proliferating cells, with NMNAT converting NMN directly to NAD+; the Preiss–Handler route proceeds through NaMN and NaAD, with NADSYN1 catalyzing the terminal amidation, and enters downstream of NAMPT; *de novo* synthesis from tryptophan is capped at the ACMSD branch point. Nicotinamide is itself a branch point, partitioning between NAMPT-dependent salvage and NNMT-dependent methylation to 1-methylnicotinamide. **b** Distinct NMNAT isoforms and SLC25A51-mediated transport generate pools that are not freely interchangeable. **c** Four enzyme families consume NAD+ stoichiometrically and release nicotinamide, coupling consumption to synthesis. **d** NAPRT status, rather than NAD+ concentration, determines whether NAMPT blockade is pharmacologically achievable [16, 17, 45]

Mammalian cells generate NAD+ by three routes that converge on a common product but differ in substrate, regulation, and tumor distribution [8, 44]. Salvage runs through NAMPT to nicotinamide mononucleotide (NMN) and then to NAD+ through the nicotinamide mononucleotide adenylyltransferases (NMNATs); the Preiss–Handler route enters downstream of NAMPT through NAPRT. Figure 1 carries the enzymes, intermediates, and compartments of all three routes, and the relative flux each supplies. *De novo* synthesis from tryptophan proceeds through QPRT, with low baseline flux that is inducible by inflammatory signaling [45]. NAMPT inhibition suppresses only the first route, so its effect on total cellular NAD+ depends on the capacity of the other two (Fig. 1).

This imbalance is consequential for T-cells and NK cells. In melanoma and head and neck squamous cell carcinoma, CD8+ + T cells in the TME show reduced oxidative phosphorylation (OXPHOS) relative to CD4 + subsets including regulatory T-cells (Tregs), indicating a shift toward glycolysis [46]. Single-cell metabolic maps show glycolysis and OXPHOS usage varying by intratumoral niche [46]. The T-cell receptor (TCR)-PLCgamma-TUB-NAMPT axis sustains intracellular NAD+ in CD8 + tumor-infiltrating lymphocytes (TILs), and its disruption lowers glycolytic capacity, mitochondrial function, ATP production, and effector function [29]. In a K562 model in vivo, nicotinamide restored NAD+ in CAR-T-cells, increased TIL density, and extended survival [29]. In hepatocellular carcinoma (HCC) patient tissues, NAMPT expression and NAD+ levels are decreased in tumor-infiltrating NK cells [47]; reduced NK NAMPT promoted tumor growth, and NMN restored anti-tumor NK responses in HCC models [47]. Impaired mitochondrial function drives TIL dysfunction [48], and nicotinamide riboside (NR) enhances mitochondrial function and improves anti-PD-1 response [48]. NAD+ salvage likewise supports NK cell mitochondrial metabolism and cytotoxicity, and NAD+ insufficiency drives NK dysfunction that NMN rescues *in vivo* [47]. Immune cell NAD+ biology is therefore a determinant of whether checkpoint blockade translates into durable cytotoxicity.

Intratumoral NAMPT expression and high NAD+ correlate with susceptibility to anti-PD-L1 therapy in GBM [28, 46, 49]. NAD/NAMPT also modulates myeloid programs and macrophage polarization, influencing immunosuppression and antigen presentation [3]. In chronic lymphocytic leukemia (CLL), extracellular NAMPT promotes M2 macrophage polarization, driving progression while suppressing nonmalignant T-cell proliferation [50]. In pancreatic ductal adenocarcinoma, interferon signaling depletes NAD+ by upregulating PARP9, PARP10, and PARP14 [51], so innate immune activation impairs anti-tumor immunity through NAD+ consumption. NAMPT also drives the proangiogenic shift of tumor-associated neutrophils (TANs), promoting vascularization and proliferation [52]. The proangiogenic function of NAMPT contributes to the metastatic capacity of tumor cells [53]. Targeting TRAF3IP2 in glioblastoma reduces tumor vascularization through inhibition of multiple proangiogenic mediators including VEGF, IL6, ANGPT2, and FGF2, linking inflammatory signaling to neoangiogenesis in the TME [54].

### NAMPT as the rate-limiting oncogenic node

NAMPT is overexpressed across solid and hematologic malignancies including colorectal, ovarian, breast, gastric, hepatocellular, pancreatic (PDAC), GBM, lymphoma, and melanoma [4, 14–16, 26, 55, 56]. A meta-analysis of 2737 patients across 15 tumor types confirmed elevated NAMPT as an independent negative prognostic factor for overall survival [14, 57]. NAMPT supports NAD+ synthesis and acts as an extracellular ligand that drives oncogenic signaling and stromal reprogramming [15, 55]. NAMPT regulates the transcription factor E2F2 and thereby inhibitor of differentiation (ID) protein transcription, which sustains self-renewal in GBM stem cells [58]. In melanoma, NAMPT drives proliferation and blocks p53-mediated apoptosis through the E2F2/SIRT1 axis [59]. In colorectal cancer, NAMPT correlates with elevated SIRT1, PARP1, and TGF-beta signaling [49]. BRAF inhibitor–resistant melanoma cells become more motile and invasive [60], upregulate NAMPT, and undergo apoptosis when it is targeted [56]. Three pharmacologically separable pools exist. Intracellular NAMPT (iNAMPT) is the target of all catalytic inhibitors to date (FK866, CHS828, and OT-82). Extracellular NAMPT (eNAMPT) functions predominantly as a cytokine-like damage-associated molecular pattern (DAMP) through Toll-like receptor 4 (TLR4), promoting M2 polarization and NF-κB activation [50, 55, 61]. Catalytic inhibitors do not antagonize eNAMPT cytokine functions, which are mediated through protein–protein interactions outside the catalytic domain, explaining the incomplete efficacy of first-generation agents.

TRAF3-interacting protein 2 (TRAF3IP2) is a master regulator of malignant signaling in glioblastoma, whose targeting remodels the tumor microenvironment and inhibits tumor growth [62]. Inhibition of TRAF3IP2 attenuates metabolic activity in glioblastoma cells by decreasing NAMPT expression and mTOR signaling, reducing NAD synthesis and glycolytic function [63]. This regulatory axis extends to triple-negative breast cancer, where TRAF3IP2 inhibition similarly decreases NAMPT and disrupts cellular bioenergetics across tumor types [64]. TRAF3IP2 silencing regresses preformed TNBC tumors and prevents macro- and micrometastasis while disrupting tumor–mesenchymal stem cell (MSC) communication [65].

### Tripartite NAMPT pharmacological taxonomy: iNAMPT, eNAMPT, and vNAMPT

NAD+ is distributed across nuclear (NMNAT1), cytosolic (NMNAT2), and mitochondrial (NMNAT3) pools with distinct signaling outputs [66]. Using genetically encoded biosensors, Hoyland et al. showed that the mitochondrial compartment buffers depletion in other pools through SLC25A51-mediated redistribution and that exhausting this reserve triggers bioenergetic failure disproportionate to the fractional loss of total NAD+ [66]. This compartmentalization has direct consequences for tumor immunology. CD8 + TILs exhibit disturbed mitochondrial dynamics, impaired cristae structure, and diminished OXPHOS capacity [29, 48]. These phenotypes may reflect exhaustion of the mitochondrial buffer as tumor CD38 activity and lactate-driven acidification deplete cytoplasmic NAD+ in TILs. That yields a testable prediction: therapies restoring mitochondrial NAD+ specifically, through NMNAT3-targeted delivery or SLC25A51 modulation, should outperform systemic precursor supplementation. It has not been tested.

Vesicle-associated NAMPT (vNAMPT) is the third pharmacologically separable pool and the least well characterized. Yoshida et al. established that eNAMPT circulates predominantly within extracellular vesicles in mice and humans, with free protein the minor fraction, is internalized by recipient cells and raises NAD+ biosynthesis in target tissues including primary hypothalamic neurons; adipocyte-specific NAMPT overexpression increased vesicular eNAMPT, improved physical activity, and extended lifespan [67]. This separates two properties that are otherwise conflated. Free eNAMPT signals as a cytokine-like DAMP through TLR4 without requiring catalytic activity, whereas vesicle-encapsulated NAMPT retains and delivers enzymatic function, protected from proteolysis, into recipient cytoplasm. The two forms therefore differ in target, pharmacology, and consequence.

Macrophage-derived eNAMPT drives an acetylated STAT3-DIRAS2 axis in triple-negative breast cancer, enhancing stemness through bidirectional crosstalk between macrophages and malignant cells [68]. Directionality matters: if tumor-derived vesicles export NAMPT to stromal and myeloid populations while depleting the extracellular precursor pool available to lymphocytes, one secretory process would enrich the immunosuppressive compartment and impoverish the effector compartment. This extends the metabolic ecology described above but remains a hypothesis, and the contribution of vesicular transfer to total tumor NAD+ flux has not been measured in any system. Three recent studies bear on vesicular transfer. Yeung et al. found NAMPT enriched in small extracellular vesicles from metastatic hepatocellular carcinoma, showed that vesicular NAMPT activates TLR4-dependent NF-κB signaling to induce SLC27A4 and glycolytic flux in recipient cells, and reported that FK866 suppressed tumor growth across several *in vivo* models [69]. Jiang et al. described the reciprocal arrangement in gastric cancer, where macrophage-derived vesicles carrying NAMPT set the nicotinamide to 1-methylnicotinamide ratio in the microenvironment and thereby determine CD8 + T-cell function; autologous transfer of these vesicles restored cytotoxicity and the anti-PD-1 response [70]. Ghezzi et al. detected NAMPT and nicotinamide N-methyltransferase released both as free protein and within vesicles by BRAF inhibitor–resistant melanoma cells, tying vesicular release to acquired drug resistance [71]. The vesicular pool is therefore functional and pharmacologically addressable. Its directional role in tumors remains a hypothesis. None of these studies quantifies the contribution of vesicular transfer relative to free eNAMPT, and the prediction that follows from the hypothesis, that blocking vesicle loading should lower recipient-cell NAD+ while leaving free eNAMPT signaling intact, has not been tested.

### The CD38-CD203a-CD73 noncanonical adenosine axis as a druggable cascade

CD38-mediated NAD+ catabolism generates immunosuppressive adenosine through a noncanonical purinergic pathway. In the canonical cascade, extracellular ATP is sequentially hydrolyzed by CD39 (ecto-nucleoside triphosphate diphosphohydrolase 1) to AMP and by CD73 (ecto-5′-nucleotidase) to adenosine. In a parallel noncanonical pathway, CD38 degrades NAD+ to ADP-ribose (ADPR), CD203a (ectonucleotide pyrophosphatase/phosphodiesterase 1 (ENPP1)) converts ADPR to AMP, and CD73 dephosphorylates AMP to adenosine [72–74]. When the canonical CD39-CD73 pathway is inhibited, tumor cells exploit the CD38-initiated cascade to sustain local adenosine production [73]. Horenstein et al. showed that in multiple myeloma the bone marrow niche generates adenosine primarily from NAD+ by this route, suppressing T effector and NK cytotoxicity while expanding Tregs and myeloid-derived suppressor cells (MDSCs) [72]. All three enzymes are independently druggable, with agents approved or at a clinical stage.

**Fig. 2:**
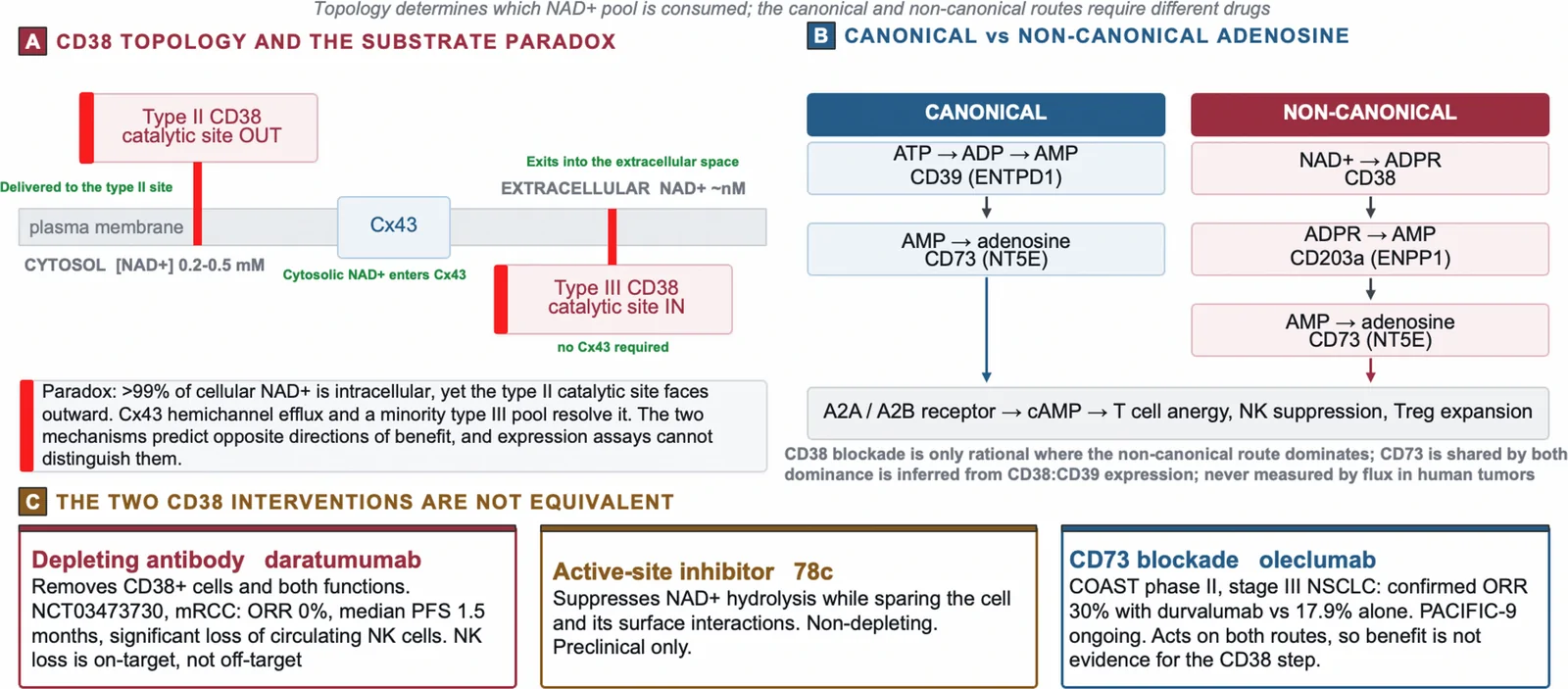
CD38 topology, the two routes to intratumoral adenosine, and separable CD38 pharmacology. **a** CD38 is a type II ectoenzyme whose catalytic domain faces the extracellular space, while more than 99% of cellular NAD+ is intracellular. Connexin 43 hemichannel efflux and a minority type III orientation resolve this separation and predict opposite directions of benefit from blockade; type III reaches cytosolic NAD+ directly and requires no transport. **b** Adenosine is generated by a canonical CD39–CD73 route from ATP, in which CD39 hydrolyzes ATP stepwise through ADP to AMP, and a noncanonical CD38–CD203a–CD73 route from NAD+; both converge on A2A and A2B receptors, but only the latter is CD38-dependent, so CD38 blockade is rational only where the noncanonical route predominates, a dominance that has been inferred from expression and never measured by flux. **c** Depleting antibodies and active site inhibitors are not interchangeable: daratumumab delivers both catalytic blockade and cytotoxic depletion, producing no objective responses and significant NK cell loss in metastatic renal cell carcinoma, an on-target consequence of that design, whereas CD73 blockade combined with PD-L1 inhibition improved a response rate in stage III non-small-cell lung cancer (NSCLC); because CD73 serves both routes, that benefit is not evidence for the CD38 step [31, 72, 75, 77]

The membrane topology of CD38 determines which NAD+ pool this cascade consumes, and the question is unresolved in a way that bears directly on therapeutic interpretation (Fig. 2). Because most cellular NAD+ is cytosolic and does not cross membranes by simple diffusion, the type II ectoenzyme shown in Fig. 2a is nominally separated from its principal substrate. The paradox has been recognized for nearly three decades [75] and has two proposed resolutions. Connexin 43 hemichannels mediate calcium-regulated efflux of intracellular NAD+ to the ectocellular catalytic site, and a minority of CD38 adopts the opposite type III orientation, accessing cytoplasmic NAD+ directly [44, 75, 76].

The therapeutic consequence is specific. If tumor CD38 depletes immune cell NAD+, it must consume NAD+ that lymphocytes exported through connexin hemichannels, making the effect a property of the interstitial compartment and dependent on hemichannel activity in the effector cell. If instead the dominant activity is type III CD38 acting on cytosolic NAD+, the depletion is cell-autonomous and tumor CD38 would principally deplete tumor NAD+. The two mechanisms predict opposite directions of benefit from CD38 blockade and are not distinguished in any oncology study cited above, all of which measure expression rather than orientation or flux. Orientation is therefore a mechanistic hypothesis with a testable consequence. Where type III CD38 predominates, blockade should raise tumor NAD+ and change lymphocyte NAD+ little; where hemichannel efflux predominates, the reverse. Neither has been measured in a tumor. Resolving this would clarify why anti-CD38 antibodies improved outcomes with checkpoint blockade in murine models yet produced no responses as monotherapy in renal cell carcinoma.

## NAD+ pathway modulation and immunotherapy: mechanistic basis and therapeutic classes

### Convergent evidence for NAD+ as a regulator of checkpoint response

**Fig. 3:**
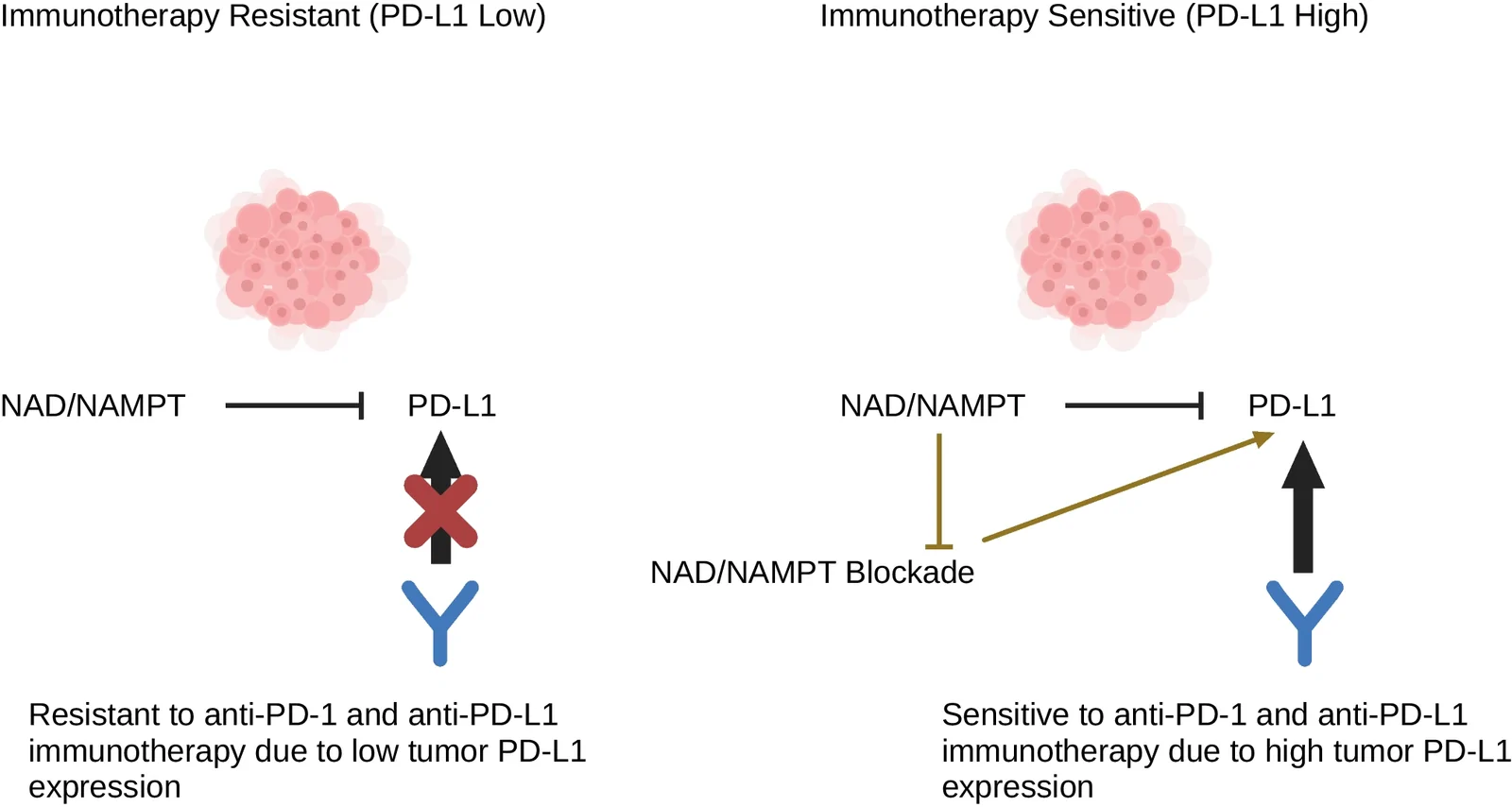
NAD/NAMPT blockade converts immunotherapy-resistant to immunotherapy-sensitive tumors. Left panel: in this model, NAD/NAMPT activity restrains PD-L1 expression, so the untreated tumor is PD-L1 low and resistant to anti-PD-1/PD-L1 immunotherapy. Right panel: pharmacological NAMPT blockade paradoxically upregulates PD-L1 through the TET1–IRF1 epigenetic axis, converting resistant tumors to a checkpoint-sensitive state. This provides the rationale for Strategy I (tumor-directed NAD+ depletion combined with checkpoint blockade) (26, 28). Figures 3 and 4 show opposite directions of the same relationship and are presented together deliberately: the direction of PD-L1 change after NAD/NAMPT manipulation differs between glioblastoma and hepatocellular models, and that discrepancy is appraised in the section on conflicting evidence

Local NAMPT inhibition in glioblastoma upregulates PD-L1, expands CD3 +, CD4 +, and CD8 + infiltrates, reduces Foxp3 + regulatory T-cells, and shifts macrophages toward an M1-like phenotype, sensitizing tumors to anti-PD-1 therapy [28]. In hepatocellular and other models, intact NAMPT-driven synthesis maintains inducible PD-L1 through a metabolic-epigenetic program involving alpha-ketoglutarate, TET activity, interferon–STAT signaling, and IRF1-dependent transcription [26]. These observations are mechanistically distinct and convergent. Opposite manipulations of the same pathway can each improve checkpoint response, in different compartments and disease states (Fig. 3).

**Fig. 4:**
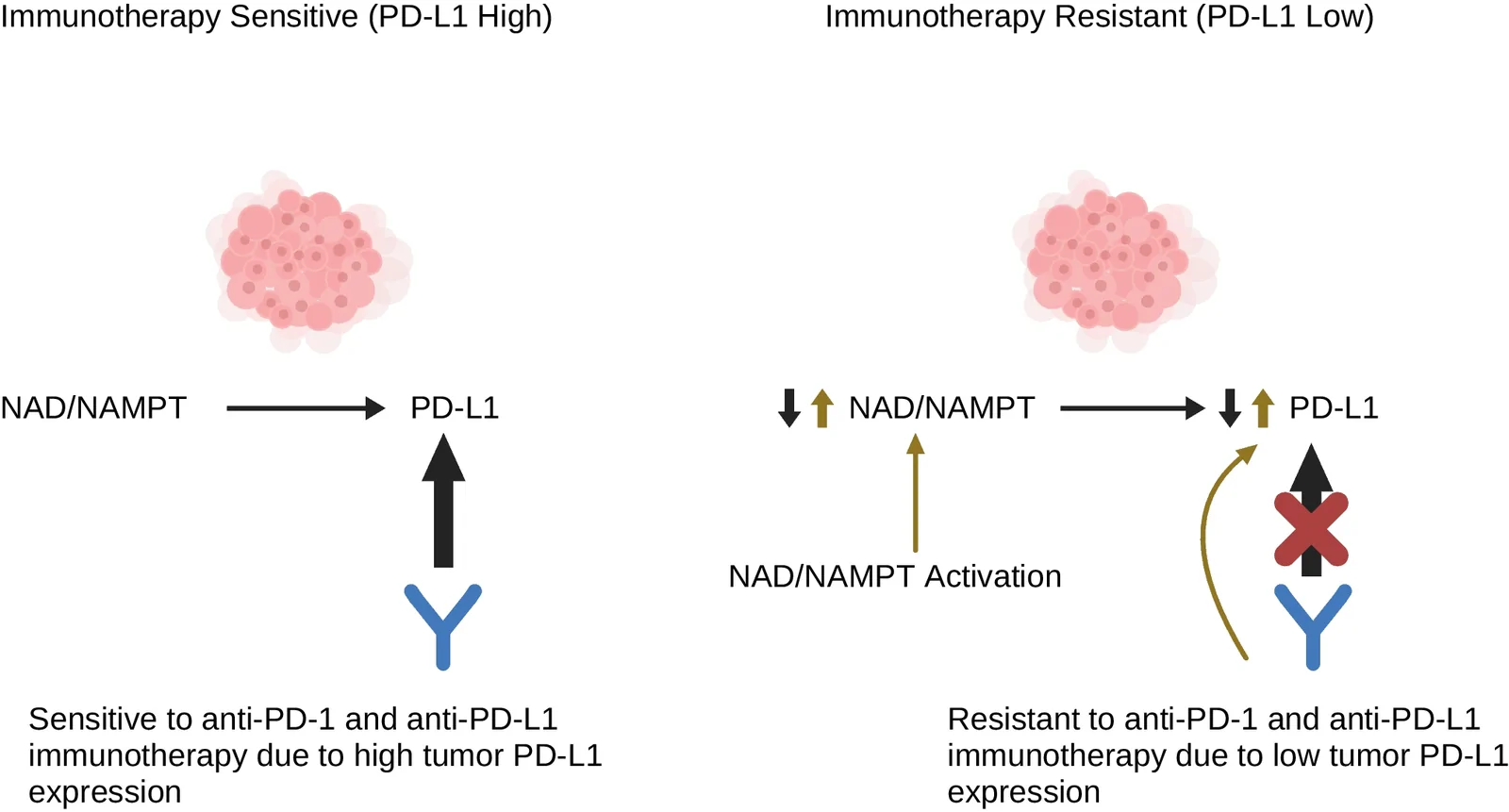
Alternative role of NAD/NAMPT in immunotherapeutic sensitivity and resistance. NAD/NAMPT activation maintains or restores immunotherapy sensitivity. Left panel: tumors with high NAD/NAMPT activity maintain elevated PD-L1 expression and are sensitive to anti-PD-1/PD L1 immunotherapy. Right panel: immunotherapy-resistant tumors with low NAD/NAMPT and low PD-L1 can be sensitized through NAD/NAMPT activation, which restores PD-L1 expression and creates susceptibility to checkpoint blockade. This provides the rationale for Strategy II (immune directed NAD+ restoration combined with checkpoint blockade) [26, 29, 49]. The directional conflict with Fig. 3 is addressed in the section on conflicting evidence

The sirtuin family (SIRT1–7) couples NAD+ availability to immune function through compartment-specific deacetylation. In the context of tumor immunity, SIRT1 suppresses pro-inflammatory gene expression via NF-κB p65 deacetylation and promotes Treg differentiation [6].

The mitochondrial sirtuins SIRT3 to SIRT5 govern oxidative phosphorylation and redox balance, and the nuclear sirtuins SIRT1 and SIRT6 restrain glycolytic reprogramming, so falling NAD+ withdraws support from both arms simultaneously [78]. These immune-specific sirtuin functions explain why NAD+ depletion in TILs is immunosuppressive beyond simple bioenergetic failure. SIRT1 downregulation with simultaneous NAMPT, PARP, and CD38 upregulation in SARS-CoV-2 infection reinforces that NAD turnover imbalance determines immunometabolic outcomes [78].

### NAD+ regulation of immune pathways beyond PD-L1

The emphasis on PD-L1 above reflects the density of the literature. NAD+ -dependent immune regulation is broader. Three further axes warrant integrated treatment.

The first is cyclic GMP-AMP synthase (cGAS) and stimulator of interferon genes (STING) innate immune sensing. Cytosolic double-stranded DNA arising from replication stress or unrepaired damage activates cGAS, generating cyclic GMP-AMP and driving STING-dependent type I interferon production. The connection to NAD+ runs in both directions and is easily misread. NAD+ depletion limits PARP-dependent single-strand break repair, increasing cytosolic DNA and enhancing STING signaling; yet sustained interferon signaling induces the NAD+ -consuming enzymes PARP9, PARP10, and PARP14, which deplete NAD+ further and, in pancreatic ductal adenocarcinoma, sensitize cells to NAMPT inhibition [51]. Innate immune activation and NAD+ depletion therefore reinforce one another. Whether the net effect favors anti-tumor immunity or exhaustion depends on stimulus duration, a temporal dimension absent from most single-timepoint designs.

Direct evidence for NAD+ -dependent STING regulation within T-cells has since been reported. Shen et al. found that nicotinamide supplementation raised T-cell NAD+ and activated phosphorylated STING and downstream interferon signaling, enhancing T-cell activation and anti-tumor function in ovarian cancer models [79]. The mechanism is specific: elevated NAD+ promoted ubiquitin-dependent degradation of SURF4, a cargo receptor mediating Golgi-to-endoplasmic-reticulum retrieval, and SURF4 loss retained STING on the Golgi, where it signals. NAD+ therefore regulates STING through subcellular trafficking, which is why transcriptional data do not predict the effect.

The direction of intervention matters here: this is a case in which raising NAD+ enhances an innate immune output within the effector compartment, which is consistent with the compartment model and provides a mechanism distinct from bioenergetic rescue. A second implication is combinatorial. The same study reported that nicotinamide supplementation amplified T-cell STING activation by PARP inhibitor-treated tumor cells, and that the combination of PARP inhibition with nicotinamide augmented T-cell anti-tumor function and restrained tumor progression [79]. This supports the NAMPT-PARP-innate immune axis proposed under therapeutic class V, with one qualification: the benefit came from raising NAD+ in the immune compartment alongside PARP inhibition of the tumor, not from depleting it in both. The three-way combination discussed below assumes the opposite direction in the immune compartment, and the tension is unresolved.

The second is macrophage polarization. NAD+ availability determines macrophage functional programming independently of its effects on lymphocytes. Minhas et al. showed that *de novo* NAD+ synthesis specifies macrophage immune function during aging and inflammation, with pathway loss producing mitochondrial dysfunction and a characteristic inflammatory phenotype [80]. Extracellular NAMPT drives M2 polarization in chronic lymphocytic leukemia through TLR4-dependent signaling, promoting disease progression and suppressing nonmalignant T-cell proliferation [50], whereas intratumoral NAMPT inhibition in glioblastoma shifts macrophages toward an inducible nitric oxide synthase (iNOS)-positive M1 phenotype [28]. These are consistent if the relevant variable is eNAMPT cytokine signaling, and they show why iNAMPT and eNAMPT require separate pharmacological consideration: an agent lowering intracellular NAD+ while leaving extracellular NAMPT signaling intact may repolarize macrophages in the wrong direction. Meng et al. add a treatment-induced route to the same endpoint. Irradiation of head and neck squamous carcinoma reprogrammed NAD+ metabolism and loaded tumor-derived extracellular vesicles with nicotinamide, which drove infiltrating macrophages toward an immunosuppressive phenotype by binding USP7 and limiting NF-κB nuclear translocation; NAMPT inhibition lowered vesicular nicotinamide and restored radiosensitivity [81]. Macrophage NAD+ programming is therefore modifiable by therapy itself, which bears on any regimen placing a NAMPT inhibitor alongside radiation.

The third is T-cell exhaustion proper. Chatterjee et al. showed that CD38 surface expression is inversely related to intracellular NAD+ in T-cells and co-expressed with PD-1 on exhausted populations; CD38-deficient T-cells had higher NAD+, increased oxidative phosphorylation and glutaminolysis, altered mitochondrial dynamics, elevated antioxidant and stem-like programs, and markedly improved tumor control on adoptive transfer [82]. CD38 therefore helps establish exhaustion, which provides the mechanistic basis for the age-associated CAR-T failure described below. Upstream, CD38 accumulation dictates age-related NAD+ decline and mitochondrial dysfunction through an SIRT3-dependent mechanism [83], placing CD38 causally at the origin of the aged lymphocyte phenotype. It also identifies a metabolic limitation of checkpoint monotherapy: PD-1 blockade does not restore mitochondrial capacity in lymphocytes that have already lost it. Two independent systems support this reading. Feng et al. showed that an interleukin-4 fusion protein acting directly on CD8 + T cells enriched functional terminally exhausted cells within tumors and improved the outcome of both adoptive transfer and checkpoint blockade; mechanistically it raised glycolytic flux and intracellular NAD+ through lactate dehydrogenase A, and the metabolic effect was indispensable for reinvigoration [84]. Raising NAD+ within the effector compartment therefore reverses exhaustion through the same redox node that lactate constrains. Huang et al. reached the same conclusion from the therapeutic direction: single-cell multiomics of patient-derived CAR-T products identified CD38 as a hallmark of exhaustion, and inhibiting CD38 enzymatic activity reversed both tonic-signaling and antigen-driven exhaustion independent of construct design, acting through combined suppression of CD38–cADPR–calcium signaling and activation of a CD38-NAD+ -SIRT1 axis [85]. This replicates the CD38-NAD+ -exhaustion link in human cells and in a clinically relevant product.

The paradox resolves once tumor-intrinsic and immune-intrinsic NAD+ pools are separated. In tumor cells, high salvage flux sustains malignant metabolism and inducible PD-L1, promoting immune evasion while creating a dependence on checkpoint blockade. In immune cells, inadequate NAD+ drives mitochondrial collapse, exhaustion, and reduced cytotoxicity. Global direction of change is the wrong variable; what matters is whether the intervention exposes a tumor-specific vulnerability or restores immune-cell fitness. Systems that omit one compartment, such as tumor-only *in vitro* models or immunodeficient mice, misclassify the direction of response (Fig. 4).

## Conflicting evidence and the strength of the compartment dependence model

The studies summarized above point in opposite directions, and the compartment dependence model should be tested against this conflict.

The clearest contradiction concerns the direction of NAD+ manipulation required to sensitize tumors to checkpoint blockade. Li et al. reported that intratumoral NAMPT inhibition in the GL261 glioblastoma model upregulated PD-L1, expanded CD3 +, CD4 +, and CD8 + infiltrates, reduced Foxp3 + Tregs, shifted macrophages toward iNOS-positive M1, and improved survival with anti-PD-1 [28]. Lv et al. reported the converse in hepatocellular and lung models: NAMPT-driven NAD+ synthesis was required to maintain inducible PD-L1, and NMN supplementation, which raises NAD+, sensitized anti-PD-L1-resistant Lewis lung carcinoma (LLC) and Pan02 tumors to checkpoint blockade [26]. Each is internally consistent and reproduced within its own system, but they cannot both generalize.

Three explanations are compatible with the data and are not equally supported. The compartment hypothesis advanced in this review holds that the direction of benefit depends on whether the rate-limiting deficit lies in tumor salvage flux or lymphocyte mitochondrial reserve. Its strongest support is the immunodeficient-versus-immunocompetent comparison of Lv et al., in which NAMPT-deficient tumors grew normally without adaptive immunity but were restrained when it was present [26], which localizes the effect to the immune compartment. The hypothesis does not, however, explain why identical manipulation of NAMPT produces opposite PD-L1 responses in glioblastoma and hepatocellular carcinoma, and that remains its principal weakness. A second explanation attributes the difference to baseline state. GL261 glioblastoma is poorly inflamed and PD-L1 low, so NAMPT inhibition raises PD-L1 from a floor and creates a target; the hepatocellular models carry inducible PD-L1 that depends on continuing salvage flux, so withdrawing NAMPT withdraws it. On this account, the starting PD-L1 and interferon tone set the direction, and compartment is secondary. A third explanation is methodological. Li et al. delivered GMX1778 intratumorally, whereas Lv et al. used genetic deletion with systemic NMN. Local saturating exposure of the tumor with relative sparing of lymphoid tissue is a different intervention from systemic manipulation, and the two experiments may not be comparable. This explanation is untested and the easiest of the three to settle. A further discrepancy is narrower but instructive. Chen et al. reported that in bladder cancer NAMPT negatively regulates PD-L1 under interferon-gamma-conditioned culture, the opposite direction to the hepatocellular data, and that NAD+ supplementation raised PD-L1 only in the absence of interferon-gamma [86]. Direction of effect therefore depends on the inflammatory context of the assay as well as on the compartment, which constrains generalization further.

These three reconciliations are hypotheses, and the distinction from what has been shown matters. The two opposing results are reproducible observations, each confirmed within its own system. The demonstrated mechanism is narrower: the TET1–IRF1 route to PD-L1 in hepatocellular models, and the loss of the growth phenotype in immunodeficient hosts. No current evidence discriminates among the three reconciliations, and none has been tested against the others in a single system. Compartment dependence is therefore a hypothesis under test in this Review, and it is held to the same standard it is applied with. A model that accommodates every result explains none.

The CD38 literature requires similar caution. CD38 acts as a tumor suppressor in prostate cancer, where overexpression depletes proliferative NAD+ pools and arrests the cell cycle [18–20], and as a mediator of acquired resistance in models where it is transcriptionally induced following PD-1 or PD-L1 blockade [21]. These are often cited together as evidence of context dependence, but they answer different questions: the prostate studies manipulate CD38 on the tumor cell and measure tumor proliferation, whereas the resistance studies measure the consequence of tumor CD38 for adjacent lymphocytes. The paradox is partly an artifact of differing readouts and conflating them overstates context dependence as a unifying principle.

A comparable caveat applies to the evidence base as a whole. Much of the mechanistic work reviewed here comes from syngeneic murine models with high baseline immunogenicity, from a few cell lines, or from ex vivo human systems lacking stromal and vascular context. Cross-model replication is uncommon, and negative results are underreported. Where clinical data exist, as for NAMPT catalytic inhibitors and anti-CD38 antibodies in solid tumors, it is less encouraging than the preclinical literature predicts. That discrepancy is treated below as a constraint on interpretation.

MYC-driven induction of PD-L1 and CD47 [87], together with interferon-induced IRF1 binding at the PD-L1 promoter [88], shows how classical oncogenic and inflammatory pathways converge on checkpoint expression through a metabolic state. Global manipulation of NAMPT or NAD+ therefore yields mixed outcomes across tumor types, because the pathway acts on a system with multiple feedback loops.

### Therapeutic class I: NAMPT catalytic inhibitors and the lessons of clinical failure

FK866 (APO866), a competitive reversible inhibitor of the NAMPT catalytic site, entered phase II for relapsed or refractory CTCL (NCT00431912). It was terminated for futility: no complete responses were observed, with dose-limiting thrombocytopenia as the principal adverse event [24]. CHS828 (GMX1778), a cyanoguanidine NAMPT inhibitor with a distinct binding mode, completed phase I dose escalation in patients with advanced solid tumors but was not advanced owing to progressive disease across dose cohorts and a toxicity profile precluding dose intensification [25]. Both programs were conceived as cytotoxic monotherapy, which is pharmacologically incoherent when NAMPT inhibition depletes NAD+ in malignant and immune cells alike. Without concurrent checkpoint blockade to exploit the PD-L1 upregulation that accompanies NAD+ depletion [26, 28], the immunosuppressive consequences may counterbalance direct anti-tumor cytotoxicity. Clinical-stage NAMPT catalytic inhibitors and their outcomes are summarized in Table 1.

**Table 1:** NAD+-directed therapeutic agents organized by pharmacological class

Agent	Class	Target(s)	Mechanism	Clinical stage	Key limitation	Ref
FK866 (APO866)	I	iNAMPT	Competitive catalytic inhibitor; NAD+ depletion	Phase II (halted): CTCL	Thrombocytopenia; no ICB combination; no biomarker selection	[24]
CHS828 (GMX1778)	I	iNAMPT	Cyanoguanidine; NAD+ depletion	Phase I (not advanced)	Progressive disease; unselected patients	[25]
OT-82	I	iNAMPT	Improved therapeutic index; reduced retinal and cardiac toxicity	Phase I: hematologic malignancies (NCT03921879)	Hematologic only; no ICB combination	[89]
Compound T8	II	iNAMPT + PD-L1	Oral dual inhibitor; IC_50_ 63 nM (PD-L1), 0.582 μM (NAMPT); oral F 78.8%	Preclinical	Murine data only	[90]
LZFPN-90	II	iNAMPT + PD-L1	Dual metabolic-immune inhibition	Preclinical	Early development	[30]
NAMPT-PROTAC	II	iNAMPT + eNAMPT	Proteasomal degradation; eliminates catalytic + cytokine functions	Preclinical	vNAMPT unaddressed	[91]
NMN + 78c	III	NAD+ pool + CD38	NAD+ repletion + CD38 NADase inhibition; CAR-T metabolic rescue	Preclinical (human ex vivo)	Not yet clinical; aged-donor specific	[92]
Daratumumab	IV	CD38	Anti-CD38 mAb; cell depletion + NAD+ catabolism blockade	FDA-approved (myeloma)	Not tested in triple-axis combination	[21]
Isatuximab	IV	CD38	Anti-CD38 mAb	FDA-approved (myeloma)	Not tested in triple-axis combination	[12, 31]
Quemliclustat	IV	CD73	Small-molecule CD73 inhibitor; terminal adenosine blockade	Phase II	Monotherapy efficacy limited	[74]
Olaparib	V (combo)	PARP1/2	PARP trapping; synthetic lethality with NAMPT inhibitors via NAD+ substrate depletion	FDA-approved (BRCA-mutant)	Not combined with NAMPT inhibitors clinically	[93]

OT-82, a structurally optimized NAMPT inhibitor engineered for improved therapeutic index with reduced retinal and cardiac toxicity, is undergoing phase I evaluation for relapsed/refractory hematologic malignancies (NCT03921879) [89]. Its design reflects partial learning: the indication is restricted to hematologic cancers, where the therapeutic window is wider, but the protocol includes no checkpoint combination.

### Pathway redundancy: NAPRT and QPRT as determinants of intrinsic resistance

Tumors with intact NAPRT expression maintain NAD+ homeostasis through the Preiss–Handler pathway during NAMPT inhibition [16]. The selective NAPRT inhibitor 2-hydroxynicotinic acid (2-OHNA) overcomes this redundancy in preclinical models [16]. NAPRT status should therefore be measured and prespecified in future NAMPT inhibitor trials. A second escape route runs through *de novo* synthesis: upregulation of QPRT, which converts quinolinic acid to nicotinic acid mononucleotide, lets tumors bypass salvage entirely by channeling tryptophan-derived carbon through the kynurenine pathway (*17*). This escape route intersects with IDO-mediated immunosuppression (therapeutic class V).

This redundancy is more consequential than its usual treatment as a caveat implies, and it is a candidate explanation for much of the failure of first-generation agents. NAPRT is silenced by promoter hypermethylation in some tumors and retained in others, a bimodal distinction. In NAPRT-proficient cells, physiologic nicotinic acid concentrations are sufficient to sustain NAD+ through the Preiss–Handler route during complete NAMPT blockade, so the achievable pharmacodynamic effect approaches zero. An unselected trial therefore contains a subgroup in which the drug cannot work at any tolerated dose, diluting any true effect toward the null. Neither the FK866 nor the CHS828 program measured NAPRT status, and both negative results are consistent with, though they do not prove, this interpretation. These claims should be kept apart. That an alternative route can carry NAD+ synthesis is a biochemical capability shown in cell systems. That it does so under NAMPT blockade is an experimentally demonstrated resistance mechanism in the models where 2-OHNA restored sensitivity [16]. That it causes treatment failure in patients is neither demonstrated nor excluded, because no trial has measured it. NAPRT status is a resistance biomarker in proposal only.

The *de novo* route imposes a second, more tractable constraint. QPRT converts quinolinic acid, the terminal product of tryptophan catabolism, to nicotinic acid mononucleotide, entering NAD+ synthesis downstream of NAMPT. Basal flux is low in most tissues but inducible, and the inducing signal is the same inflammatory environment checkpoint blockade is intended to create. This yields a testable prediction: NAMPT inhibition with checkpoint blockade should select for *de novo* upregulation as resistance, preventable by concurrent IDO1 inhibition. The prediction is untested and is the principal rationale for the combination under therapeutic class V.

### Therapeutic class II: bifunctional compounds and degradation-based platforms

Yang et al. mapped a reciprocal regulatory circuit between NAMPT and PD-L1 expression across multiple cancer cell lines [30]. Tumors with a NAMPTHighPD-L1Low phenotype carry the worst prognosis. NAMPT inhibition upregulates surface PD-L1 through SIRT-dependent deacetylation and NF-κB p65 activation, whereas PD-L1 blockade reciprocally induces NAMPT through HIF-1α-dependent glycolytic reprogramming [30].

Lv et al. demonstrated that NAMPT-mediated NAD+ synthesis promotes tumor immune evasion through upregulation of tumor cell PD-L1 [26]. NAD+ and alpha-ketoglutarate, derived from NAD+ -dependent conversion of isocitrate, activate TET expression [26], establishing the NAMPT–TET1–pSTAT1–IRF1–PD-L1 epigenetic axis. Interferon stimulation activates STAT, which recruits TET to demethylate IRF1, driving PD-L1 transcription [26]. In immunodeficient mice, NAMPT-deficient and control liver cancer cells produced tumors of similar size; in immunocompetent hosts, NAMPT-deficient cells produced smaller tumors, with increased TILs and killing activity, whereas NMN promoted growth and reduced TIL number and activation. Activation of the NAMPT/PD-L1 axis is therefore associated with poor outcome. Paradoxically, anti-PD-L1-resistant tumors (LLC lung and Pan02 pancreatic) can be sensitized when NAD+ is restored by NMN, likely through induction of tumor PD-L1. The combination of NMN and anti-PD-L1 antibody inhibited tumor progression [26]. Two subsequent studies extend the axis beyond surface expression. Lu et al. found that NAMPT-dependent NAD+ supply sustains SIRT1 activity, which deacetylates histone H3 at lysine 27 and drives PD-L1 into the nucleus in cervical cancer, identifying subcellular localization rather than abundance as the regulated variable [94]. Gao et al., using spatiotemporal multiomics across the normal-to-cancer sequence in early gastric cancer, identified a NAD-dependent immunosuppressive niche in which NAMPT mediates tumor cell interaction with fibroblasts, and showed that targeting NAMPT suppressed JAK-STAT, MAPK, and NF-κB signaling, lowered PD-L1, and delayed malignant transformation [95]. The second places associated at the earliest disease stage so far were examined.

Intratumoral delivery of the NAMPT inhibitor GMX1778 upregulates PD-L1 expression and increases surface protein levels in GBM cells [28]. Combining NAMPT inhibition with anti-PD-1 improves survival in the GL261 mouse model of GBM [28]. NAMPT inhibition increased CD3 +, CD4 +, and CD8 + T cells and decreased Foxp3 + Tregs [28]. iNOS-positive CD68 + macrophages (M1 polarization, anti-tumor activity) were increased, while Arg-1-positive macrophages (anti-inflammatory, pro-tumoral function) were decreased. One of seven treated animals achieved complete tumor clearance, a single animal result that is consistent with NAMPT inhibitor monotherapy failure being overcome through immunological combination [28]. Pharmacological blockade of the STAT/TET1 axis promotes survival and synergizes with chemotherapy in a mouse model of acute myeloid leukemia (AML) [96].

Dual NAMPT/PD-L1 small-molecule inhibitors: T8 and LZFPN-90. Bifunctional small molecules exploit the reciprocal NAMPT–PD-L1 axis. Compound T8, an orally bioavailable dual NAMPT/PD-L1 inhibitor (IC50: 63 nM against PD-L1, 0.582 micromolar against NAMPT; oral bioavailability 78.8% in mice), achieved a tumor growth inhibition (TGI) of 40.1% in B16-F10 melanoma, compared with 28.2% for anti-PD-L1 antibody monotherapy [90]. LZFPN-90, designed on the basis of the reciprocal regulatory loop, demonstrated superior TGI relative to anti-PD-L1 monotherapy in the same model [30]. Both are preclinical, and the margins over antibody monotherapy, while consistent, are modest and derive from one syngeneic model.

PROTAC-mediated NAMPT degradation. NAMPT-directed proteolysis-targeting chimeras (PROTACs) recruit E3 ubiquitin ligase complexes to NAMPT, inducing polyubiquitination and proteasomal degradation [90, 91]. Because the whole protein is eliminated, degradation ablates iNAMPT catalytic activity and eNAMPT DAMP/cytokine functions together. Of the modalities in development, PROTAC degradation is the only one that could address both pools concurrently. This rests on a pharmacological logic: published NAMPT PROTACs have been characterized for target degradation and tumor growth inhibition, and their effect on eNAMPT cytokine signaling *in vivo* is unreported.

### Consolidated overview of NAMPT-directed pharmacological strategies

NAMPT-directed approaches are distributed across several therapeutic classes above and are drawn together here so the landscape for this single target can be assessed as a whole. These strategies are distinguished by which NAMPT pool they engage.

Competitive catalytic inhibition, represented by FK866 and CHS828, occupies the substrate-binding site of iNAMPT and has been tested clinically without success, for reasons of trial design and patient selection discussed above. Structurally optimized catalytic inhibition, represented by OT-82, retains the mechanism while improving the therapeutic index through reduced retinal and cardiac toxicity and remains in phase I in hematologic malignancies. Bifunctional inhibition, represented by compound T8 and LZFPN-90, combines catalytic NAMPT blockade with PD-L1 antagonism in a single molecule, exploiting the reciprocal regulation of the two targets; both are preclinical. Targeted degradation by NAMPT-directed PROTACs eliminates the protein and is the only strategy addressing the noncatalytic functions of eNAMPT, though its effect on the extracellular pool has not been demonstrated. Bypass-pathway inhibition, principally NAPRT blockade with 2-hydroxynicotinic acid, addresses intrinsic resistance, leaves NAMPT untouched, and applies only to NAPRT-proficient tumors. Pathway modulation through upstream regulators, including TRAF3IP2 inhibition, lowers NAMPT expression indirectly and has shown activity in glioblastoma and triple-negative breast cancer models [63, 64].

Five of the six strategies engage iNAMPT alone, leaving eNAMPT and vNAMPT effectively undrugged. None has been evaluated clinically with checkpoint blockade, despite reciprocal NAMPT–PD-L1 regulation being the most reproducible immunological finding in the field. A rational development program would address these first.

### Therapeutic class III: NAD+ precursor immunorestorative therapy

Vardhana et al. showed that impaired mitochondrial OXPHOS limits self-renewal of antigen-experienced T-cells under chronic stimulation and that NR enhanced anti-PD-1 responses [97]. Yu et al. showed that disturbed mitochondrial fission–fusion dynamics in CD8 + TILs reinforces the exhaustion program and that NR partially restored network integrity and cytolytic function [48]. Both are *in vivo* murine results, and they have been reproduced across independent models. No NAD+ precursor has been tested clinically as an immunorestorative agent in cancer, so this class rests on reproducible preclinical evidence with no clinical support. Global targeting or enhancement of NAD+ pathways can therefore produce dual effects through differential impact on effector and tumor cells.

Age-associated NAD+ decline as a modifiable determinant of CAR-T efficacy. Hope et al. showed that CAR-T cells from aged mice have NAD+ -dependent mitochondrial dysfunction, with loss of stem-like markers (CCR7hiCD45RAhi), diminished mitochondrial membrane potential, and impaired anti-tumor function *in vivo* (*92*). CD38 was elevated on CD8 + T cells from aged donors, and CD38hi subpopulations showed reduced TMRM fluorescence consistent with mitochondrial depolarization. Published single-cell RNA-sequencing data from anti-CD19 CAR-T cohorts confirmed that chronological age and NAD+ pathway gene signatures both correlate with response [92]. Ex vivo NMN with 78c, a selective inhibitor of the CD38 NADase domain, restored NAD+/NADH ratios, rescued basal and maximal oxygen consumption on Seahorse analyses, and re-established stem-like immunophenotypes in aged CAR-T cells [92].

Roughly 75% of patients with cancer are over 65, the group in which CAR-T efficacy declines most steeply, and age-associated NAD+ decline is pharmacologically correctable at least ex vivo. Neither NMN nor 78c has an established dose, schedule, or exposure target for ex vivo conditioning, and raising NAD+ during manufacture risks driving differentiation away from the stem-like phenotype that confers persistence. The correlation between age, NAD+ gene signatures, and response is retrospective and does not establish causation, since age covariates with prior therapy, marrow reserve, and disease biology. The data justify a formal preclinical program with *in vivo* endpoints, followed if successful by a manufacturing feasibility study with metabolic and phenotypic correlates. Direct incorporation into clinical protocols is premature.

### Therapeutic class IV: adenosinergic axis targeting and the triple-blockade proposal

CD38 rises in tumors treated with anti-PD-1/PD-L1 antibodies and promotes resistance; dual CD38 and PD-L1 inhibition restored anti-tumor immunity [21]. Preserving immune-cell NAD+ therefore matters directly to checkpoint resistance. Daratumumab and isatuximab, FDA-approved anti-CD38 antibodies for multiple myeloma, are available for adenosinergic combinations in solid tumors without new drug development.

The triple-axis CD38/CD73/checkpoint blockade strategy. Suppressing the NAD+ -derived adenosinergic program would require simultaneous intervention at three nodes: (i) anti-CD38 antibodies (daratumumab and isatuximab) to prevent NAD+ catabolism and ADPR generation; (ii) CD73 inhibitors (quemliclustat and oleclumab; phase II) to block terminal adenosine generation at the convergence point of both canonical and non-canonical pathways; and (iii) anti-PD-1/PD-L1 checkpoint inhibitors to release the coinhibitory brake on T-cell effector function. All three classes contain agents that are FDA-approved or in late-stage development. Availability should not be mistaken for evidence of benefit, and the clinical data impose substantial constraints on this proposal.

The most directly relevant clinical evidence is discouraging. Campbell et al. conducted a pilot study of single-agent daratumumab in muscle-invasive bladder cancer and treatment-refractory metastatic renal cell carcinoma (NCT03473730) [31]. Treatment was safe, with no toxicity events in either cohort, and target engagement was confirmed: CD38-expressing cells were depleted in the circulation and within tumor tissue. No objective responses were observed in the renal cell cohort, and median progression-free survival was 1.5 months (95% confidence interval, 1.1–1.8 months). A single pathologic complete response occurred among eight patients with bladder cancer, a cohort too small to interpret. Immune monitoring demonstrated significant reductions in circulating natural killer cells in both cohorts.

Scheduling is distinct from patient selection and has not been specified in any proposal combining anti-CD38 antibodies with checkpoint blockade. Daratumumab both inhibits the ectoenzyme and depletes CD38-expressing cells, and circulating natural killer cells fell substantially in both cohorts [31]. Because natural killer cells drive antibody-dependent cytotoxicity and the early response to checkpoint inhibition, concurrent administration risks removing an effector population just as checkpoint blockade is meant to mobilize it. Sequential and concurrent dosing predicts different outcomes. Sequential dosing with anti-CD38 given far enough ahead to allow natural killer reconstitution, preserves the enzymatic rationale while limiting overlap with the effector-dependent phase. Concurrent dosing maximizes adenosine suppression but accepts the depletion. A trial that does not specify which schedule it is testing cannot interpret a negative result, and neither has been evaluated. The constraint does not extend to the CD73 component, where the terminal step of adenosine generation is blocked without cell depletion [74].

Evidence for the CD73 component is stronger than the evidence for the CD38 component. In the randomized phase II COAST study, oleclumab added to durvalumab consolidation in unresectable stage III non-small-cell lung cancer raised the confirmed objective response rate to 30.0% from 17.9% with durvalumab alone and prolonged progression-free survival without new safety signals (*32*). This supports adenosine axis blockade with PD-L1 inhibition as a tractable strategy, though it tested two agents, was not powered for definitive comparison, and did not stratify by CD38 or CD73 expression. The phase III PACIFIC-9 trial evaluates the same combination and will provide the first adequately powered assessment. In the neoadjuvant setting, durvalumab with oleclumab produced higher major pathologic response rates than durvalumab alone in resectable non-small-cell lung cancer [77], so the benefit is not confined to consolidation. On present evidence the defensible next step is not a three-agent trial but a biomarker-selected two-agent study establishing whether CD38 blockade adds anything to CD73 inhibition in tumors with a documented noncanonical adenosine phenotype.

### Therapeutic class V: mechanistically rationalized synergistic combinations

NAMPT plus PARP inhibitors: synthetic lethality with immunogenic consequences. PARPs consume NAD+ as a substrate for poly(ADP-ribosyl)ation during DNA single-strand break repair. PARP and NAMPT inhibitors are therefore synthetic lethal-like: NAMPT inhibition depletes the NAD+ substrate pool required for PARP-dependent repair, and PARP inhibition prevents repair of the damage that NAD+ insufficiency precipitates [98]. Heske et al. demonstrated synergistic anti-tumor activity of combined NAMPT and PARP inhibition in Ewing sarcoma models [93]. The immunological extension runs through cGAS-STING: unrepaired damage from PARP inhibition generates cytoplasmic double-stranded DNA that activates cGAS-STING, driving type I interferon production and PD-L1 upregulation [79]. A three-way combination of NAMPT inhibition, PARP inhibition, and anti-PD-1 would bring together metabolic synthetic lethality, innate immune activation, and adaptive checkpoint release. The evidence behind its three steps is uneven. The NAMPT–PARP pairing has *in vivo* support in Ewing sarcoma [93]; the cGAS-STING step is carried over from PARP inhibitor biology [79]; the full combination has not been tested in any model.

NAMPT plus IDO inhibitors: closing the tryptophan-NAD+ resistance loop. The *de novo* NAD+ biosynthetic pathway from tryptophan proceeds through the kynurenine pathway, catalyzed by the rate-limiting enzymes indoleamine 2,3-dioxygenase (IDO1) and tryptophan 2,3-dioxygenase (TDO). IDO1 satisfies the same criteria: tryptophan depletion from the TME activates the amino acid starvation sensor GCN2 in T-cells, suppressing proliferation, while kynurenine engages the aryl hydrocarbon receptor (AhR) to drive Foxp3 + Treg differentiation [99]. The kynurenine pathway ultimately converges on NAD+ through quinolinic acid and QPRT [7]. This creates a pharmacological paradox: IDO-mediated immunosuppression simultaneously replenishes tumor NAD+ through *de novo* synthesis, a defined resistance mechanism against NAMPT inhibitors [17]. Co-targeting IDO1 and NAMPT would, on this reasoning, block immunosuppressive tryptophan catabolism and close the *de novo* escape route. The combination rests on mechanistic rationale alone. It has not been tested, and the QPRT-dependent escape it is designed to prevent has not been demonstrated in a tumor under NAMPT blockade.

In circadian NAD+ regulation, the clock proteins CLOCK/BMAL1 directly regulate NAMPT transcription, producing NAD+ oscillation with approximately 24-h periodicity (*100*). Circadian disruption, common in cancer, deregulates NAMPT expression and this oscillation, potentially sustaining NAD+ elevation in tumor cells. SIRT1 feeds back on CLOCK/BMAL1, closing a NAD+ -sirtuin-clock loop [100]. Timing NAMPT inhibitor administration to tumor NAD+ troughs may improve efficacy while reducing systemic toxicity. No chronotherapeutic study of a NAMPT inhibitor has been reported.

## Integrated translational framework: biphasic therapeutic strategy

The scheme below is hypothesis-generating. It arranges current evidence into a biphasic, context-dependent scheme stratified by tumor immunometabolic phenotype, and no element of it has been tested prospectively:

- Strategy I: tumor-directed NAD+ depletion. In tumors with salvage dependence, low immune fitness, and inducible PD-L1, NAMPT inhibition (class I or II agents, alone or with NAPRT or PARP inhibitors) depletes tumor NAD+, upregulates PD-L1 through the TET1–IRF1 and NF-κB axes, repolarizes macrophages from M2 to M1, reduces intratumoral Treg density, and sensitizes to concurrent checkpoint blockade. Candidate contexts, assigned from the phenotype of preclinical models and not from compartment-resolved measurement, include GBM, melanoma, and BRAF inhibitor-resistant malignancies.
- Strategy II: immune-directed NAD+ restoration. Where T-cell or NK cell exhaustion dominates, class III agents (NMN, NR, or ex vivo NAMPT support) rescue effector mitochondrial bioenergetics and cytolytic capacity. Candidate contexts, on the same basis, include HCC, NSCLC, and tumors with high CD38 driving immune-cell NAD+ depletion. NMN with a CD38 inhibitor (78c) preconditioning during ex vivo CAR T-cell manufacture is the most readily testable of these [92].

Both strategies require checkpoint antibodies to translate PD-L1 dynamics into a benefit. Without them, tumor-directed depletion risks immune escape through unopposed PD-L1 upregulation, and immune-directed restoration risks fueling tumor proliferation through non-selective supplementation. Assigning a given tumor to one arm is the weakest element of the scheme. The assignment rests on the phenotype of model systems, and it would need compartment-resolved NAD+ measurement in human tumors before it could function as a clinical decision rule. No such measurement exists.

### Limits of compartment-selective modulation

**Fig. 5:**
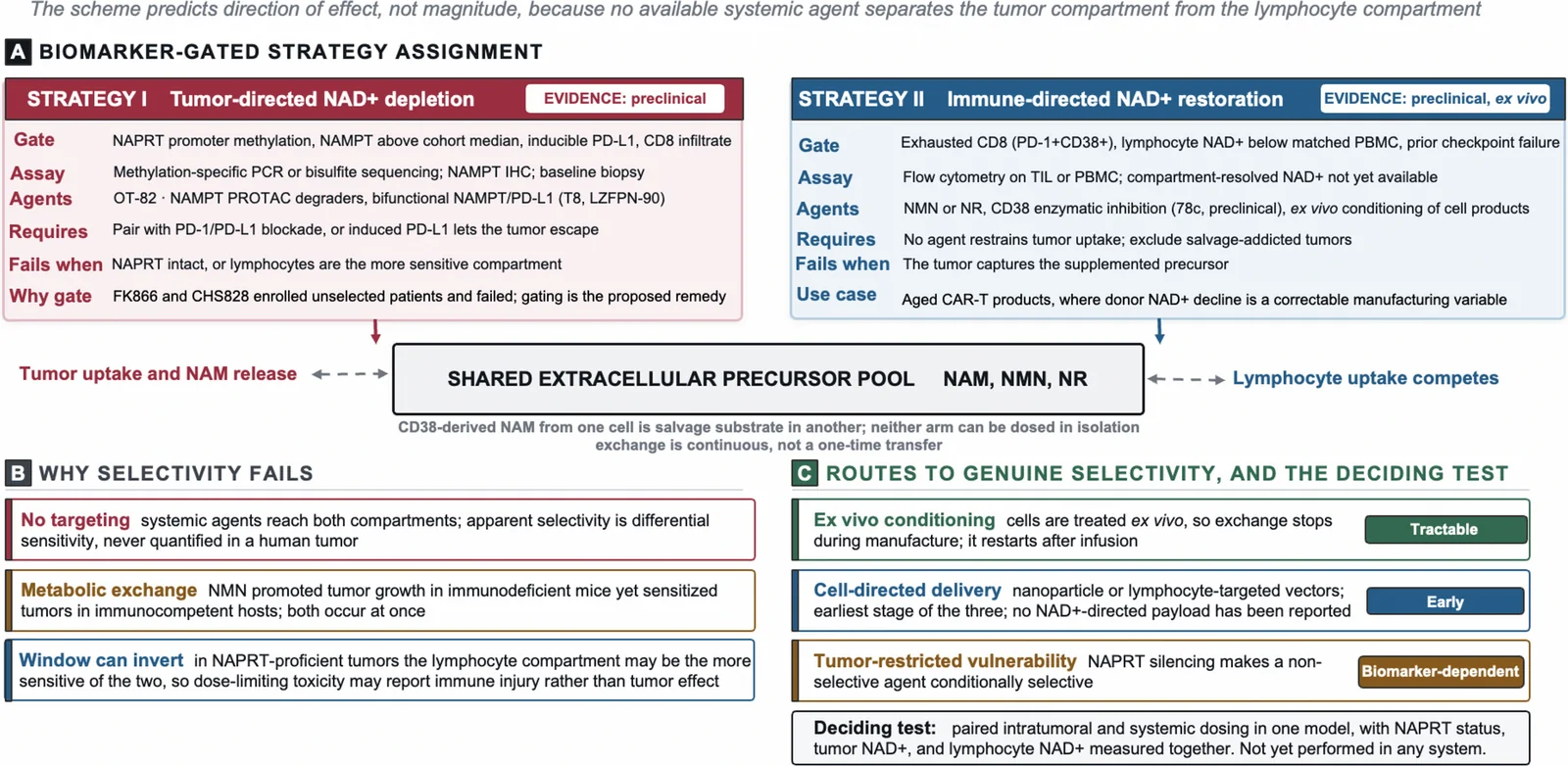
Compartment-resolved therapeutic logic and the constraints on its execution. **a** The scheme assigns tumor-directed depletion and immune-directed restoration to biomarker-defined states, with anthe assay required for each gate, the candidate agents, and the conditions under which each arm is predicted to fail. Both arms rest on preclinical evidence, and compartment-resolved NAD+ measurement in patients is not yet available. Malignant and immune populations draw on a shared extracellular precursor pool, so neither arm can be dosed in isolation. **b** Three constraints prevent genuine compartment selectivity with available systemic agents. **c** Ex vivo conditioning suspends exchange during manufacture, though it resumes after infusion; cell-directed delivery is early, and NAPRT silencing renders a non-selective agent conditionally selective [26, 39, 56, 62, 88, 100]

The biphasic scheme is a useful organizing device, but its central assumption, that tumor and immune NAD+ pools can be manipulated independently, is met by no currently available agent (Fig. 5).

Systemically administered small molecules distribute to both compartments [44]. NAMPT catalytic inhibitors do not discriminate between a malignant cell and a tumor-infiltrating lymphocyte, and neither do NAD+ precursors, which are taken up by whatever cell expresses the relevant transporters. Any apparent selectivity therefore arises from differential sensitivity: tumor cells with silenced NAPRT and high salvage flux are more vulnerable to NAMPT inhibition than lymphocytes retaining alternative routes, and this margin is the real basis of the therapeutic window. That margin is narrow, has not been quantified in any human tumor, and would be expected to collapse in NAPRT-proficient tumors, in which the lymphocyte compartment might be the more sensitive of the two. This inverts the intended effect and is a plausible contributor to the toxicity observed in early trials.

Metabolic exchange further blurs the compartments (Fig. 5b). Nicotinamide released by CD38-mediated hydrolysis in one cell is a substrate for salvage in another, across tumor, stromal, myeloid, and lymphoid populations alike. Supplementation intended for lymphocytes necessarily raises precursor availability for malignant cells, and the finding that NMN promoted tumor growth in immunodeficient mice while sensitizing tumors to checkpoint blockade in immunocompetent hosts [26] shows both effects occurring at once, with the net outcome set by which dominates. This constrains the biphasic model: it predicts a direction of effect and says nothing about magnitude (Fig. 5).

Four intervention classes follow from the scheme above: compartment-selective NAD+ modulation with checkpoint blockade; CAR-T products carrying a NAMPT construct; ex vivo NMN treatment of autologous T-cells; and T-cell-directed delivery of nanoformulated NMN or NAMPT-promoting gene therapy. The defensible question is not whether NAD+ modulation is pro-tumor or anti-tumor, but which compartment, pathway branch, and immune state are being manipulated.

### Biomarker-guided patient stratification

PD-L1 status alone is inadequate for stratification because the pathway is inducible and can reflect either checkpoint susceptibility or active immune suppression. A more informative framework integrates tumor NAMPT and NAPRT expression by immunohistochemistry and RNA sequencing; CD38 and CD73 co-expression on tumor and immune cells by multiparameter flow cytometry; baseline and interferon-gamma-inducible PD-L1 status; CD8 + TIL density and macrophage M1:M2 polarization ratio by multiplex immunofluorescence and spatial transcriptomics; circulating eNAMPT concentrations by ELISA; and sirtuin activity profiles (SIRT1, SIRT3, and SIRT6) as surrogates for compartment-specific regulation. Chronological age and NAD+ pathway gene signatures should be independent stratification variables, given the impact of age-associated NAD+ decline and CD38 upregulation on immune-cell fitness and CAR-T response [92]. The objective is state conversion, shifting a tumor from immune-excluded or metabolically buffered toward checkpoint-responsive or immune-restored (Table 2). These markers are not of one kind, and the distinction governs how each can be used. Tumor NAMPT expression, the NAD+ pathway gene signature, and CD8 + TIL density are prognostic: they associate with outcome independently of NAD+ -directed treatment. TIL density has also been examined as a predictor of checkpoint blockade response, without being qualified as one. Circulating eNAMPT, mitochondrial NAD+ status in TILs, and the M1:M2 ratio are pharmacodynamic: they report that an agent has engaged its pathway, and on their own they say nothing about who should receive it. NAPRT status, interferon-gamma-inducible PD-L1, and CD38/CD73 co-expression are predictive in form, since each is proposed to identify patients in whom a specific agent will or will not work. None of these has been qualified as a predictive biomarker in a prospective trial. A selection rule such as NAMPTHigh/NAPRTLow is therefore a hypothesis to be tested, and it is offered here as the design a trial should adopt and not as a criterion ready for clinical use.

**Table 2:** Proposed biomarker stratification panel for NAD+-targeted precision immunotherapy. No entry has been qualified as a predictive biomarker in a prospective trial; the therapeutic guidance column states hypotheses to be tested

Biomarker	Assessment method	Clinical implication	Therapeutic guidance	Drug class	Ref
Tumor NAMPT expression	Immunohistochemistry; RNA-seq; circulating eNAMPT (ELISA)	Prognostic. High NAMPT: negative prognosis; proposed to predict PD-L1 inducibility upon NAMPT inhibition	High: strategy I (class I/II + ICB); low: strategy II (class III + ICB)	I, II, III	[14, 26, 86]
NAPRT expression	IHC; RNA-seq	Predictive in form, unqualified. NAPRT-proficient: intrinsic resistance to NAMPT inhibitor monotherapy	NAPRT + : add 2-OHNA; NAPRT-: NAMPT inhibitor alone	I	[16]
PD-L1 (baseline + IFN-gamma-inducible)	IHC (22C3/28–8); flow cytometry	Predictive in form, unqualified. Low baseline/high inducibility: proposed optimum for NAMPT inhibitor-induced PD-L1 + ICB	Low/inducible: strategy I; high: strategy II	I, II, III	[26, 88]
CD38/CD73 co-expression	Flow cytometry; multiplex IHC	Predictive in form, unqualified. High: active noncanonical adenosine axis; proposed ICB resistance marker	Triple-axis: anti-CD38 + anti-CD73 + ICB	IV	[21, 72]
Patient age; NAD+ gene signature	Clinical record; scRNA-seq NAD+ metabolism score	Prognostic. Age >65: CD38 upregulation; NAD+ decline impairs CAR-T/TIL fitness	NMN + CD38i ex vivo preconditioning for CAR-T manufacturing	III	[92]
CD8+ TIL density; M1:M2 ratio	Multiplex IF; spatial transcriptomics	Prognostic and pharmacodynamic. Low TILs/high M2: immunosuppressive TME phenotype	Strategy II (immune restoration) + ICB	III, IV	[50, 80]
Mitochondrial NAD+ status	^13^C-MRS; genetically encoded NAD+ biosensors	Pharmacodynamic. Low mitochondrial NAD+ in TILs: bioenergetic exhaustion	NMNAT3-targeted or SLC25A51-modulated delivery	III	[48, 66]

## Discussion

The evidence reviewed here does not support treating NAMPT as a generic metabolic enzyme. NAD+ turnover regulates checkpoint plasticity, immune cell persistence, macrophage polarization, and treatment response through compartment-specific mechanisms. It supports a model in which NAD+ functions as a metabolic immune checkpoint in the restricted sense defined above***:*** perturbing its production or consumption can reprogram whether anti-tumor immunity is metabolically possible.

Tumor NAD+ sufficiency maintains inducible PD-L1 and other survival programs, creating both immune evasion and checkpoint targetability, while NAD+ supplementation enhances T-cell function and anti-PD-1 activity in selected immune-exhausted settings [101]. NAD+ -targeted therapies [26] can increase radiosensitivity and may be particularly attractive when DNA damage, PARP dependence, or replication stress already shapes tumor vulnerability [102]. Maintaining NAD+ pools in CD8 + TILs prevents exhaustion and sustains cytotoxicity, so NAD+ modulation must be matched to immune context as well as tumor type [97]. Raising NAMPT/NAD+ can also confer resistance to chemoradiation [102].

## Future directions

Resolving how NAD+ control differs between malignant and immune cells requires spatial, metabolic, and single-cell approaches that measure cell-state-specific flux, but bulk abundance will not answer it. The unresolved questions are which compartmental NAD+ pools determine functional collapse in exhausted lymphocytes, how salvage redundancy is distributed across tumor genotypes, and when extracellular NAD+ catabolism becomes the dominant barrier to checkpoint response.

Immune-restorative strategies using NAD+ precursors or engineered cell products should be evaluated only where lymphocyte mitochondrial insufficiency has been documented. Cell therapy is the most tractable setting because NAD+ sufficiency can be manipulated ex vivo before reinfusion. Manufacturing protocols incorporating NMN, NR, or controlled SIRT1 activation could extend persistence and cytotoxicity in solid tumors, where chronic antigen exposure erodes function. Nanoparticle or gene-based delivery could restrict metabolic intervention to the tumor niche and avoid systemic toxicity.

Real-time metabolic imaging and circulating NAD+ metabolite panels may serve as noninvasive markers of therapeutic engagement, complementing the tissue panel in Table 2.

The framework further requires a biomarker-selected comparison of CD73 inhibition with and without CD38 blockade in adenosine-high tumors; a randomized comparison of NAMPT inhibitor plus anti-PD-1 versus anti-PD-1 alone in biomarker-selected NAMPTHighNAPRTLow populations; prospective testing of NMN/CD38 inhibitor preconditioning in CAR-T manufacture for patients over 65; characterization of vNAMPT cargo loading and vesicle-directed therapeutics; and companion diagnostic panels integrating these variables.

The single most constraining experiment remains undone: paired intratumoral and systemic administration of the same NAMPT inhibitor in one model, with NAPRT status, tumor NAD+, and lymphocyte NAD+ measured together. Until then, the compartment model rests on inference from studies never designed to test it.

## Conclusion

NAD+ and NAMPT support cancer cell metabolic fitness and shape immune evasion, and these roles are separable by compartment. Monotherapy against this axis has failed repeatedly, and the trials that failed were designed without immune endpoints, without checkpoint combination, and without measuring the resistance pathway. Whether the target survives a trial that corrects those three omissions is the open question, and it has not yet been asked.

## Data Availability

No datasets were generated or analyzed during the current study.

## Abbreviations

ADPR: ADP-ribose
AML: Acute myeloid leukemia
BRAF: v-Raf murine sarcoma viral oncogene homolog B1
CAF: Cancer-associated fibroblast
CAR-T: Chimeric antigen receptor T cell
cGAS-STING: Cyclic GMP-AMP synthase-stimulator of interferon genes
CLL: Chronic lymphocytic leukemia
CTCL: Cutaneous T cell lymphoma
DAMP: Damage-associated molecular pattern
ELISA: Enzyme-linked immunosorbent assay
eNAMPT: Extracellular nicotinamide phosphoribosyltransferase
ENPP1: Ectonucleotide pyrophosphatase/phosphodiesterase 1
GBM: Glioblastoma
HCC: Hepatocellular carcinoma
HIF-1α: Hypoxia-inducible factor 1-α
ICB: Immune checkpoint blockade
IDO1: Indoleamine 2,3-dioxygenase 1
IHC: Immunohistochemistry
iNAMPT: Intracellular NAMPT
IRF1: Interferon regulatory factor 1
LLC: Lewis lung carcinoma
MDSC: Myeloid-derived suppressor cell
MRS: Magnetic resonance spectroscopy
MSC: Mesenchymal stem cell
NAD+: Nicotinamide adenine dinucleotide
NAMPT: Nicotinamide phosphoribosyltransferase
NAPRT: Nicotinic acid phosphoribosyltransferase
NF-κB: Nuclear factor kappa-light-chain-enhancer of activated B cells
NK: Natural killer
NMN: Nicotinamide mononucleotide
NMNAT: Nicotinamide mononucleotide adenylyltransferase
NQO1: NAD(P)H quinone oxidoreductase-1
NR: Nicotinamide riboside
NSCLC: Non-small-cell lung cancer
OXPHOS: Oxidative phosphorylation
PARP: Poly(ADP-ribose) polymerase
PD-1: Programmed cell death protein 1
PD-L1: Programmed death-ligand 1
PDAC: Pancreatic ductal adenocarcinoma
PROTAC: Proteolysis-targeting chimera
QPRT: Quinolinate phosphoribosyltransferase
SCLC: Small cell lung cancer
scRNA-seq: Single-cell RNA sequencing
SIRT: Sirtuin
SLC25A51: Solute carrier family 25 member 51
TAN: Tumor-associated neutrophil
TCA: Tricarboxylic acid
TDO: Tryptophan 2,3-dioxygenase
TET1: Ten-eleven translocation 1
TGI: Tumor growth inhibition
TIL: Tumor-infiltrating lymphocyte
TLR4: Toll-like receptor 4
TME: Tumor microenvironment
TMRM: Tetramethylrhodamine methyl ester
TNBC: Triple-negative breast cancer
TRAF3IP2: TRAF3-interacting protein 2
Treg: Regulatory T cell
vNAMPT: Vesicle-associated NAMPT
